# The research on the treatment of primary immunodeficiency diseases by hematopoietic stem cell transplantation: A bibliometric analysis from 2013 to 2022

**DOI:** 10.1097/MD.0000000000033295

**Published:** 2023-03-31

**Authors:** Siqi Hu, Shixia Xu, Wei Lu, Yingjian Si, Ya Wang, Zhenlan Du, Yi Wang, Zhichun Feng, Xiangfeng Tang

**Affiliations:** a Faculty of Pediatrics, the Chinese PLA General Hospital, Beijing, China; b Institute of Pediatrics, the Seventh Medical Center of PLA General Hospital, Beijing, China; c National Engineering Laboratory for Birth Defects Prevention and Control of Key Technology, Beijing, China; d Beijing Key Laboratory of Pediatric Organ Failure, Beijing, China; e Department of Pediatrics, Eden Hospital, Beijing, China; f Department of Hematology and Transplantation, Faculty of Pediatrics, the Chinese PLA General Hospital, Beijing, China; g Department of Children’s Internal Medicine, Faculty of Pediatrics, the Chinese PLA General Hospital, Beijing, China.

**Keywords:** bibliometrics, CiteSpace, hematopoietic stem cell transplantation, primary immunodeficiency diseases, publications, VOSviewer

## Abstract

Hematopoietic stem cell transplantation (HSCT) is curative in patients with primary immunodeficiency syndrome. The safety and efficacy of HSCT as a therapeutic option for primary immunodeficiency diseases (PID) have been studied by many research groups. The purpose of our study was to perform a bibliometric analysis of research on HSCT for the treatment of PID, to assess research trends in this field, and note future research priorities. The Web of Science Core Collection (WOSCC) was used to identify relevant publications. VOSviewer and CiteSpace software were used to analyze bibliometric parameters, such as yearly records, authors, grouped authors, countries, institutions, categories and keywords. There are 602 relevant records for the last decade (2013–2022). The top 5 productive authors and high-quality paper journals are listed. Reference co-citations analysis demonstrated recent research trends were “inborn errors of immunity,” “gene editing,” and “enteropathy.” Research on HSCT for the treatment of PID has increased rapidly in the last decade, and bibliometrics are valuable for researchers to obtain an overview of hot categories, academic collaborations and trends in this study field.

## 1. Introduction

Primary immunodeficiency disease (PID)—also called primary immune disorders/primary immunodeficiency/inborn errors of immunity—refers to a group of disorders characterized by the hypofunction or absence of a part of the immune system, which predisposes affected patients to various infections.^[1]^ PIDs can also lead to malignancy, inflammation and autoimmunity. To date, approximately 500 different types of disorders have been genetically identified by researchers,^[2]^ and this number continues to grow with advances in diagnostic techniques and increases in early diagnosis.^[3]^

Hematopoietic stem cell transplantation (HSCT) involves providing healthy hematopoietic stem cells to patients with depleted or dysfunctional bone marrow.^[4,5]^ The first successful allogeneic bone marrow transplant using marrow from a sibling occurred in 1968 in a 5-month-old male patient with lymphopenic immunological deficiency.^[6]^ Since then, the number of autologous and allogeneic stem cell transplants has increased worldwide. It was reported that over 8000 allogenic transplants for blood and bone marrow disorders were performed in the United States in 2016, with autologous transplants outnumbering allogenic transplants.^[4]^ Numerous studies have shown that HSCT is the definitive treatment for children with rare primary immunodeficiency disorders.^[7]^

Bibliometrics, which is a quantitative analysis, provides quantitative insights into academic literature.^[8,9]^ It can be used for a number of things in the preaward phase and is adopted to describe the trends and structure of knowledge in a specific field of research.^[10]^ Recently, bibliometric analysis has been used to provide intelligent insights into a large number of biomedical areas.^[8,9,11–14]^

In recent decades, there have been an increasing number of studies on the treatment of PID by HSCT. In this study, a bibliometric and visualization analysis of publications on HSCT and PID were utilized to track research hotspots and frontier trends. To the best of our knowledge, there are no studies that demonstrate a summary of HSCT for PID, reflect the generalization of the research in this field and note the central point of foreground exploration research.

## 2. Materials and methods

### 2.1. Search strategy

In Octuber 2022, we performed a literature search using web of science core collection (WoSCC) (Clarivate Analytics). The database is the world leading citation database and it contains records of articles from the most influential journals worldwide. The database can provide subject retrieval and define the research field to minimize invalid retrieval and make the retrieval more precise.^[15,16]^ The search terms were Topic = “(hematopoietic stem cell transplantation) or (Bone Marrow Transplantation)” AND “(primary immunodeficiency diseases) or (inborn errors of immunity).” The WoSCC database was extensively searched for relevant data from 2013 to 2022 (Jan. 2013 and Oct. 2022). The only language that was allowed was English. The following document types were included: articles, reviews, proceedings, editorial materials, and letters. Our study does not require ethical approval because the data used in the study was obtained from the relevant publications in the WoSCC.

### 2.2. Bibliometric analysis

Output search results: Export all records including search results and cited references from WoSSC in plain text format (*.txt). CiteSpace 6.1.R3 (64-bit) Basic (Drexel University, Philadelphia, PA) and VOSviewer (Version 1.6.18, Center for Science and Technology Studies of Leiden University)^[17]^ were used to locate co-cited publications, countries, institutions, journals, authors, keywords, and network features of “keyword bursts,” as well as to visualize the results. In this descriptive study, categorical variables are expressed as numbers (percentages).

## 3. Results

### 3.1. Annual outputs

**Figure 1.**
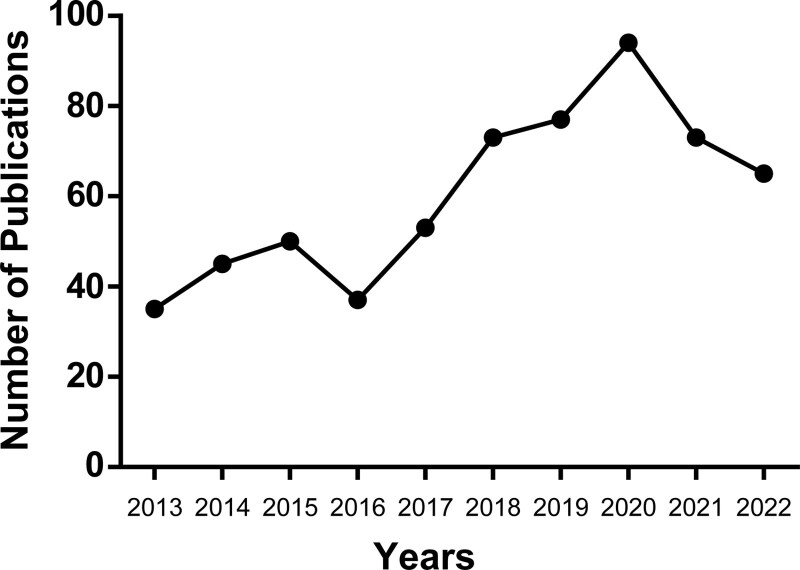
The number of publications in the research area per year from 2013 to 2022 (The number of publications collected in 2022 is incomplete).

There are 602 related research documents, including 414 articles, 167 review articles, 9 Meeting Abstracts, 6 Early Access, 5 Editorial Materials, 5 Letters, 4 Proceedings Papers, 3 Book Chapters, and 1 Reprint. The annual publication outputs are shown in Figure 1. The number of publications collected in 2022 was incomplete.

### 3.2. Authors and paper citation

**Figure 2.**
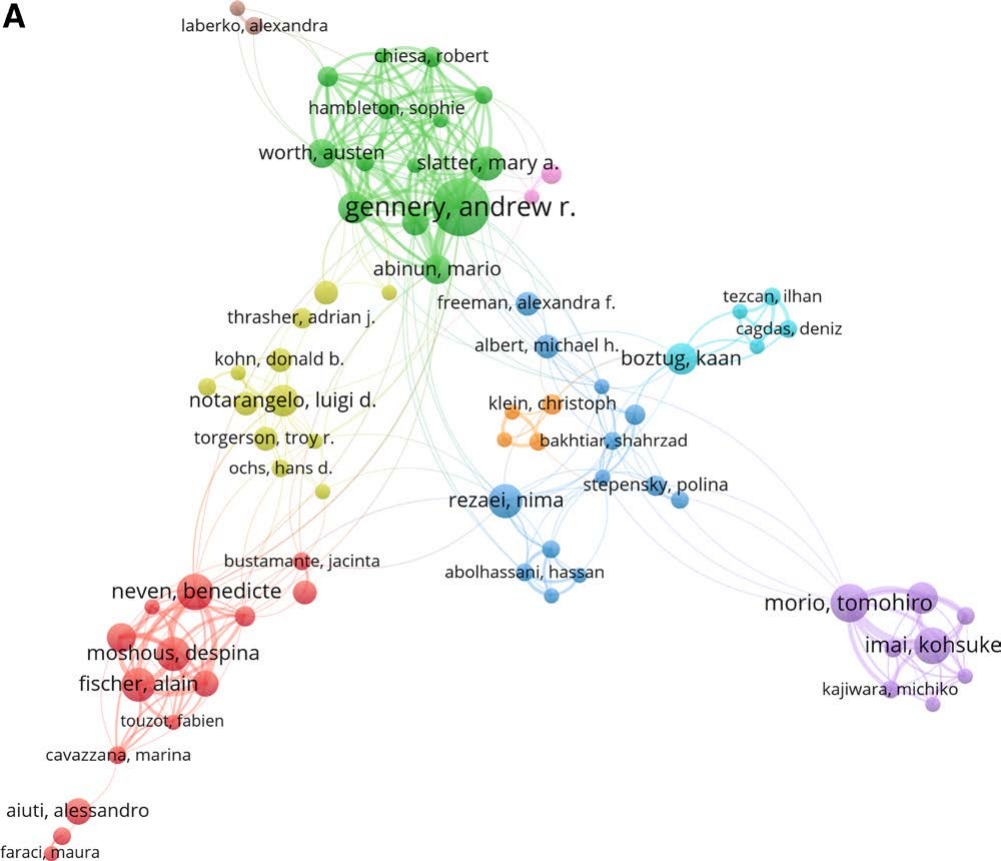
Co-authorship analysis of authors by VOSviewer. The co-authorship map of authors indicates the authors that cooperate in the field of HSCT and PID. HSCT = hematopoietic stem cell transplantation, PID = primary immunodeficiency diseases.

The top 5 productive authors were Gennery, Andrew R. (40 publications), Slatter, Mary A. (26 publications), Torgerson, Troy R. (18 publications), Notarangelo, Luigi D. (17 publications) and Neven, Benedicte (17 publications). Centrality is defined for each node in the network. It measures the likelihood of an arbitrary shortest path in the network passing through the node.^[18]^ However, the centrality scores calculated by citespace were all low, indicating less cooperation among authors. In terms of paper citation, the top 5 authors cited are Gennery, Andrew R. (463 citations), Kohn DB (212 citations), Veys, Paul (217 citations), Neven, Benedicte (192 citations), and Chiesa, Robert (172 citations) (Fig. 2).

### 3.3. Countries/regions and institutions

**Figure 3.**
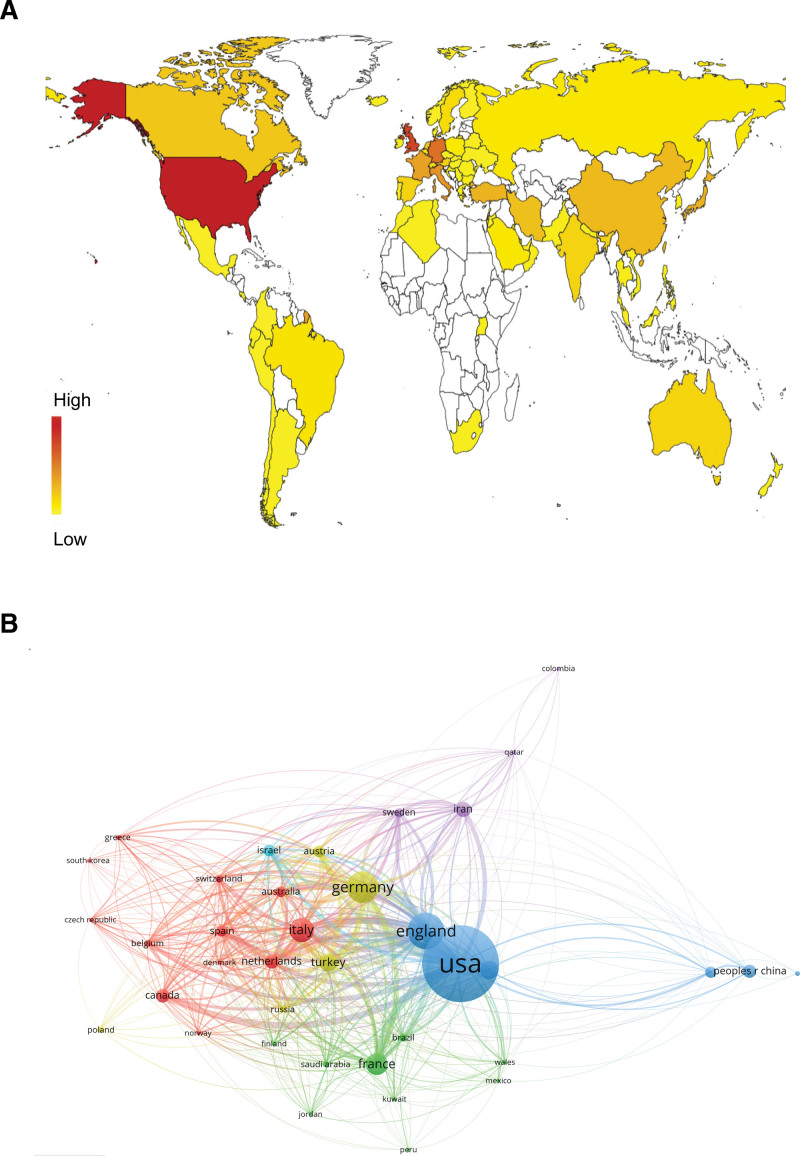
Co-authorship analysis of countries/regions. (A) Geographical distribution of publications (Note: no data available for regions not colored in the map). (B) Co-authorship analysis of countries/regions by VOSviewer. The map shows the network of co-authorship links between countries.

**Figure 4.**
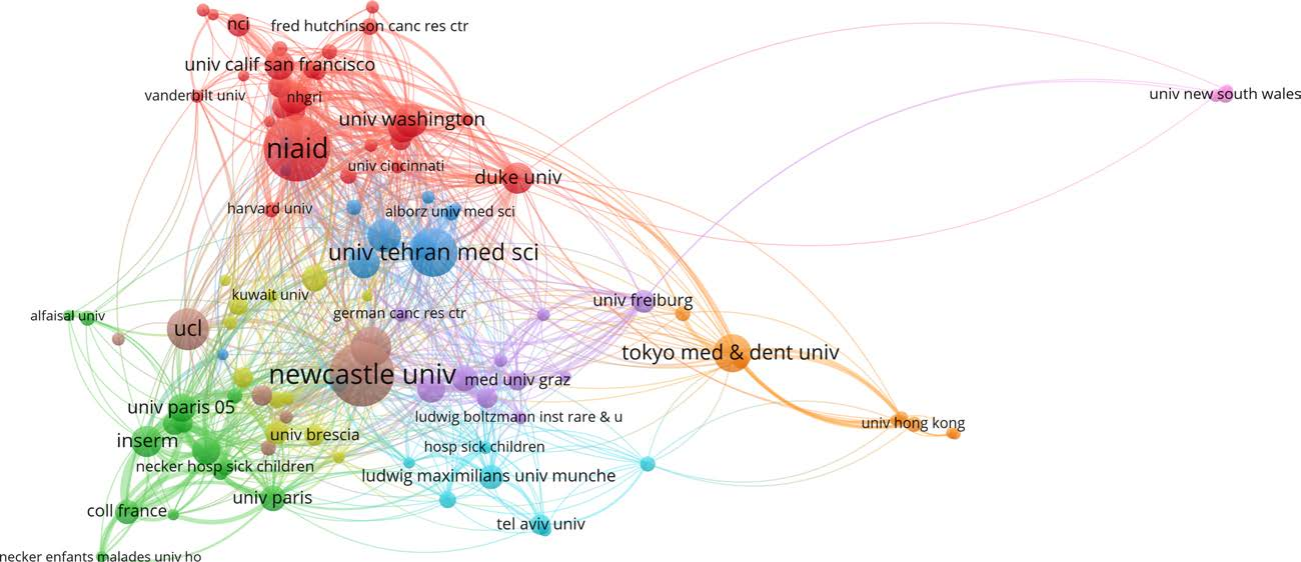
Co-authorship analysis of institutions by VOSviewer. nci: National Cancer Institute. fred hutchinson canc res ctr: Fred Hutchinson Cancer Research Center. uni calif san francisco: University of California, San Francisco. vanderbilt univ: Vanderbilt University. nhgri: National Human Genome Research Institute Home. unvi Washington: University of Washington. univ new south wales: The University of New South Wales. NIAID: National Institute of Allergy and Infectious Diseases. univ Cincinnati: University Of Cincinnati. duke univ: Duke University. harvard univ: Harvard University. alborz univ med sci: Alborz University of Medical Sciences. univ tehran med sci: Tehran University of Medical Sciences. kuwait univ: Kuwait University. univ freibury: University of Freiburg. alfaisal univ: Alfaisal University. ucl: University College London. new castle univ: Newcastle University. med univ graz: Medical University of Graz. tokyo med & dent univTokyo Medical and Dental University. univ pairs 05: Université Paris 5. inserm: Institut national de la santé et de la recherche médicale. univ Brescia: Brescia University. ludwig boltzmann inst rare & u: Ludwig Boltzmann Institute for Rare and Undiagnosed Diseases. hosp sick children: The Hospital for Sick Children. univ hong kong: The University of Hong Kong. necker hosp sick children: Necker Hospital for Sick Children. ludwig maximilians univ munche: Ludwig-Maximilians-Universität München. univ paris: Université Paris Cité. coll France: Le Collège de France. tel aviv univ: Tel Aviv University. necker enfants malades univ ho: Neker Enfants Malades Hospital.

VOSviewer software was used to analyze the data and generate national (Fig. 3) and institutional (Fig. 4) visualization maps. A total of 80 countries/regions were identified, the top 10 productive countries/regions were the United States, England, Germany, Netherlands, Turkey, Italy, France, Spain, Sweden and Australia (Table 1). A total of 200 institutions were identified during the 10 years. The top ten productive institutions are listed in Table 2. Furthermore, relative scientific societies were analyzed in our study, and the grouped authors are shown in Table 3. The European Society for Blood and Marrow Transplantation is the most important society in the field of blood and marrow transplantation and cellular therapy

**Table 1:** The top 10 productive countries/regions.

Countries/Regions	Documents	Citations
USA	229	7210
England	99	2599
Germany	84	2202
Netherlands	34	1261
Turkey	45	1025
Italy	63	1591
France	56	1502
Spain	26	707
Sweden	21	482
Australia	25	621

**Table 2:** The top 10 productive institutions.

Organization	Documents	Citations
National Institute of Allergy and Infectious Diseases	37	1007
Newcastle University	36	750
Duke University	16	666
Boston Children Hospital	16	598
University of Washington	17	551
Children Hospital of Philadelphia	15	633
Paris Descartes University	14	602
Harvard Medical School	17	690
Great North Children Hospital	21	387
Université Paris 5	14	536

**Table 3:** Grouped authors.

Group authors	Documents
European Society for Blood and Marrow Transplantation	19
Primary Immune Deficiency Treatment Consortium	4
European Society for Immunodeficiencies	3
Centre de Référence Déficits Immunitaires Héréditaires	1
Cooperative Study Group A for Hematology	1
European Reference Networks	1
French GATA2 study group	1
Japan Society for Hematopoietic Cell Transplantation	1
Medical Advisory Committee of the Immune Deficiency Foundation	1
Moroccan Society for Primary Immunodeficiencies	1
NIHR BioResource	1
Paediatric IBD Porto Group	1
Stem Cell Transplantation for Immunodeficiencies in Europe registry	1
United States Immunodeficiency Network	1

### 3.4. Journals

**Figure 5.**
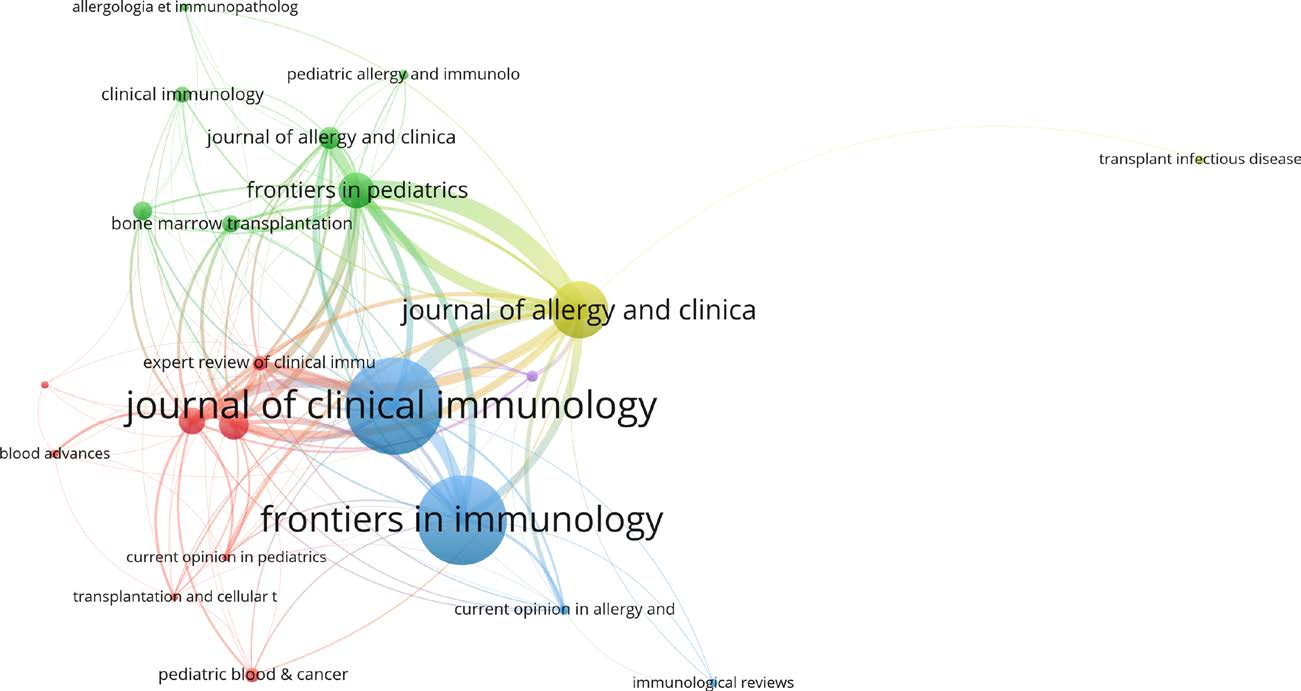
Co-authorship analysis of journals by VOSviewer. allergologia et immunopathology: Allergologia et Immunopathologia. pediatric allergy and immunolo: Pediatric Allergy and Immunology. clinical immunology: Clinical Immunology. journal of allergy and clinica: Journal of Allergy and Clinical Immunology. frontier in pediatrics: Frontiers in Pediatrics. bone marrow transplantation: Bone Marrow Transplantation. expert review of clinical immu: Expert Review of Clinical Immunology. journal of clinical immunology: Journal of Clinical Immunology. transplant infectious disease: Transplant Infectious Disease. blood advance: Blood Advances. frontier in immunology: Frontiers in Immunology. current opinion in pediatrics: Current Opinion in Pediatrics. transplantation and cellular t: Transplantation and Cellular Therapy. current opinion in allergy and: Current Opinion in Allergy and Clinical Immunology. pediatric blood & cancer: Pediatric Blood & Cancer. immunological reviews: Immunological Reviews.

We identified 149 journals that had published manuscripts in the research field. Twelve journals published over 10 documents, accounting for 296 documents or 49.2% of the total literature. Regarding the number of published documents, the relevant documents were concentrated in journals that published high-impact papers in the area of Human Immunology (Table 4). The network among the journals was analyzed using VOSviewer, as shown in Figure 5. According to the WoSCC definition, Highly Cited Papers rank in the top 1% in terms of the number of citations compared to other papers published in the same year in the same field. Table 5 shows that the 6 Highly Cited Papers are “Transplantation Outcomes for Severe Combined Immunodeficiency, 2000–2009,”^[19]^ “Update on the use of immunoglobulin in human disease: A review of evidence,”^[20]^ “Outcomes Following Gene Therapy in Patients With Severe Wiskott-Aldrich Syndrome,”^[21]^ “Phenotype, penetrance, and treatment of 133 cytotoxic T-lymphocyte antigen 4-insufficient subjects,”^[22]^ “From IPEX syndrome to FOXP3 mutation: a lesson on immune dysregulation,”^[23]^ and “Autologous Ex Vivo Lentiviral Gene Therapy for Adenosine Deaminase Deficiency.”^[24]^

**Table 4:** The top 10 productive journals.

Journals	Documents	Citations	Impact factor (2021)
*Journal of Clinical Immunology*	65	795	8.542
*Frontiers in Immunology*	60	678	8.786
*Journal of Allergy and Clinical Immunology*	38	1990	14.29
*Frontiers in Pediatrics*	24	283	3.569
*Blood*	20	591	25.476
*Transplantation and Cellular Therapy (Biology of Blood and Marrow Transplantation*)*	18	525	5.609
*Journal of Allergy and Clinical Immunology in Practice*	15	218	11.022
*Pediatric Transplantation*	13	66	1.551
*Bone Marrow Transplantation*	12	73	5.174
*Clinical Immunology*	11	213	10.19

* Formerly known as Biology of Blood Marrow Transplantation.

**Table 5:** Highly cited papers.

Title	Author (s), yr	Journal	Citations
Transplantation Outcomes for Severe Combined Immunodeficiency, 2000–2009	Pai, SY et al 2014	*New England Journal of Medicine*	407
Update on the use of immunoglobulin in human disease: A review of evidence	Perez, EE et al 2017	*Journal of Allergy and Clinical Immunology*	296
Outcomes Following Gene Therapy in Patients with Severe Wiskott-Aldrich Syndrome	Abina, SHB et al 2015	*Jama-Journal of the American Medical Association*	238
Phenotype, penetrance, and treatment of 133 cytotoxic T-lymphocyte antigen 4-insufficient subjects	Schwab, C et al 2018	*Journal of Allergy and Clinical Immunology*	199
From IPEX syndrome to FOXP3 mutation: a lesson on immune dysregulation	Bacchetta, R et al 2018	*Annals of the New York Academy of Sciences*	173
Autologous Ex Vivo Lentiviral Gene Therapy for Adenosine Deaminase Deficiency	Kohn, DB et al 2021	*New England Journal of Medicine*	40

In terms of the number of citations, highly-cited papers are concentrated in authoritative journals, including “*JOURNAL OF ALLERGY AND CLINICAL IMMUNOLOGY*,” “*JOURNAL OF CLINICAL IMMUNOLOGY*,” “*FRONTIERS IN IMMUNOLOGY*,” “*BLOOD*” “*NEW ENGLAND JOURNAL OF MEDICINE*,” and “*BIOLOGY OF BLOOD AND MARROW TRANSPLANTATION*” (Table 6).

**Table 6:** The top 10 journals cited

Journals	Documents	Citations	Impact factor (2021)
*Journal of Allergy and Clinical Immunology*	38	1990	14.29
*Journal of Clinical Immunology*	65	795	8.542
*Frontiers in Immunology*	60	678	8.786
*Blood*	20	591	25.476
*New England Journal of Medicine*	3	565	176.079
*Biology of Blood and Marrow Transplantation*	18	525	5.609
*Immunological Reviews*	6	370	10.983
*Frontiers in Pediatrics*	24	283	3.569
*Jama-The Journal of the American Medical Association*	1	238	157.335
*Journal of Allergy and Clinical Immunology in Practice*	15	218	11.022

**Figure 6.**
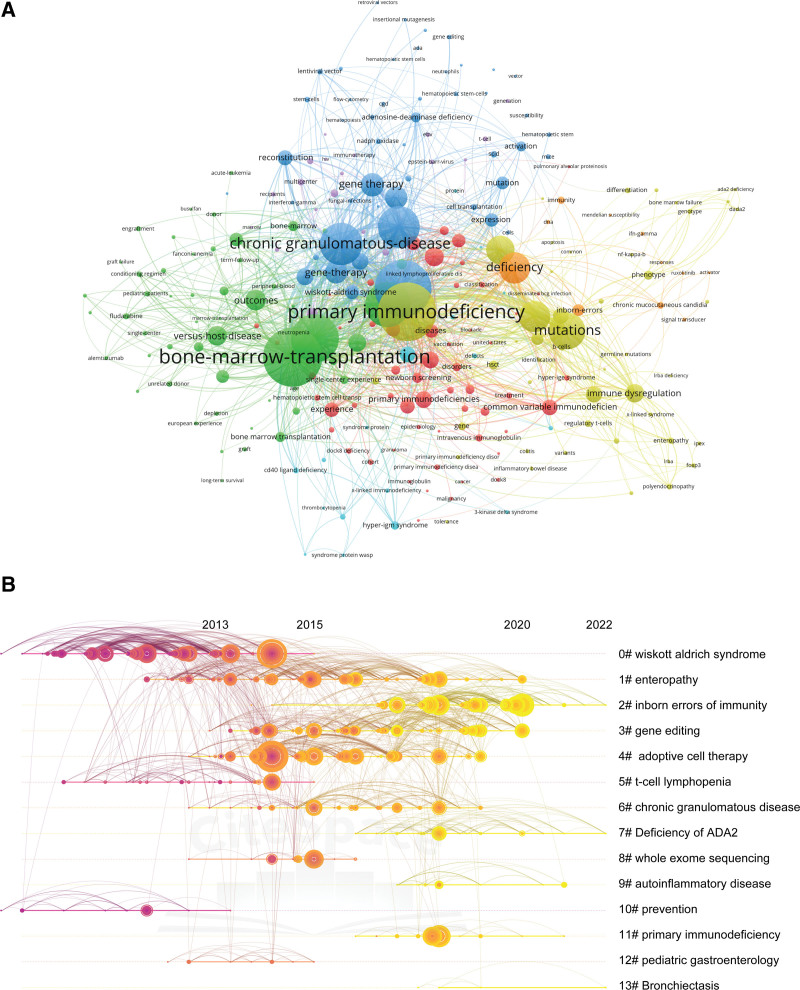
Co-authorship analysis of keywords. (A) Co-occurrence analysis of keywords by VOSviewer. (B) Timeline view of RCA determined by Citespace. The emergence time point and time span of 13 clusters are shown. RCA = reference co-citation analysis.

Web of Science Categories were assigned at the journal level. All items in a journal were assigned to the Web of Science Category of that journal.^[25]^ There were 39 Web of Science Category in our study (Table 7). The category changed from year to year as shown in Table S1 (see Table S1, http://links.lww.com/MD/I680, Supplemental Content, which illustrates the category changed from year to year). “Pediatrics” is the highly ranked category in this study, demonstrating HSCT represents the gold standard consolidation treatment for a number of pediatric patients. In addition, VOSviewer was used to conduct a clustering analysis and create a network map for co-occurring keywords. A total of 2538 keywords were identified by importing the complete records of the 602 documents. The top ten keywords are listed in Table 8. With a threshold of ≥ 5 keyword occurrences, 256 keywords were included in the network map (Fig. 6A). Next, CiteSpace was used to analyze reference co-citations, which can mirror the relationship between publications by analyzing the patterns and trends of co-citation.^[12]^ We used a timeline view to visualize the tendency of every keyword in the clusters, which are ordered from the smallest to the largest according to the number of publication co-citations.^[12]^ Figure 6 shows that the evolution of the research topics can be examined over time. Recent research trends from 2019 to 2022 included “inborn errors of immunity,” “gene editing,” and “enteropathy.”

**Table 7:** The frequency distribution of categories.

Categories	Documents	Categories	Documents
Immunology	323	Endocrinology Metabolism	4
Hematology	118	Pathology	4
Pediatrics	101	Biochemical Research Methods	3
Allergy	94	Clinical Neurology	3
Transplantation	57	Dermatology	3
Oncology	38	Education Scientific Disciplines	3
Medicine Research Experimental	28	Obstetrics Gynecology	3
Genetics Heredity	27	Public Environmental Occupational Health	3
Infectious Diseases	23	Rheumatology	2
Medicine General Internal	17	Chemistry Multidisciplinary	1
Biotechnology Applied Microbiology	16	Critical Care Medicine	1
Biophysics	12	Dentistry Oral Surgery Medicine	1
Cell Biology	11	Environmental Sciences	1
Pharmacology Pharmacy	11	Health Care Sciences Services	1
Microbiology	9	Medical Laboratory Technology	1
Multidisciplinary Sciences	8	Mycology	1
Gastroenterology Hepatology	6	Nutrition Dietetics	1
Surgery	6	Tropical Medicine	1
Biochemistry Molecular Biology	5	Virology	
Cell Tissue Engineering	5		

**Table 8:** The top 10 keywords.

Keywords	Occurrence
Bone-marrow-transplantation	146
Primary immunodeficiency	144
Stem-cell transplantation	139
Chronic granulomatous-disease	100
Hematopoietic stem cell transplantation	108
children	115
Severe combined immunodeficiency	98
Mutations	86
Deficiency	71
Disease	71

## 4. Discussion

Many scientometric analyses have been conducted in the medical field by using VoSviewer, HistCite and CiteSpace. A number of bibliometric analyses of research are on immunodeficiency, many of which focus on human immunodeficiency virus.^[26–30]^ Some bibliometric analyses have also been published on HSCT.^[31,32]^ In this study, we provide an overview of studies on HSCT for the treatment of PID.

PIDs are inherited diseases caused by deficiencies in the immune system responses and functions. PIDs are characterized by high susceptibility to autoimmunity, immune dysregulation, opportunistic infections, and malignancies.^[33]^ The first HSCT reported for PID was performed in 1968.^[34,35]^ Before 2013, HSCT was mainly used to treat malignant diseases and thalassemia in children, most of which were reported as cases. In the last 10 years, with the improvement of donor selection, conditioning regimens, gene therapy and the implication of new immunosuppressive drugs, HSCT has made rapid progress in the treatment of immunodeficiency diseases. Therefore, we mainly analyzed changes in the past decade. HSCT is an established curative treatment for many patients with PIDs. Donor selection, graft manipulation, conditioning and treatment of complications have improved incrementally, with survival and cure rates reaching 90% for some specific diseases.^[36,37]^ In the last 5 years, the number of patients with PID was increased results from advances in genetic diagnoses. In addition, use of reduced toxicity conditioning, increased use of alternative donors, and new approaches to graft engineering have significantly improved the outcome of HSCT for PID. Therefore, the number of published scientific research on the treatment of PID by HSCT increased significantly since 2016.^[38]^

The frequency distribution of publications in journals demonstrates that relevant publications were concentrated in journals that published papers in the area of human immunology. Our findings suggest that high-impact-factor journals are more frequently co-cited because of landmark publications.

The United States is the major driver for the treatment of PID by HSCT. The publication output in the US could be attributed to coordinated and integrated policies, programs and activities at different levels.^[39,40]^ National institute of allergy and infectious diseases has published many documents in this field, and it conducts and supports research to understand, treat, and prevent infectious and allergic diseases.

We were interested in the subject most frequently employed by authors when publishing in journals. Our study demonstrated that research in this field had experienced a typical process, from basic medicine to clinical applications. Of the 6 Highly Cited Papers, 2 were on gene therapy.^[21,24]^ The use of allogeneic hematopoietic stem cells for the treatment of genetic blood cell disorders has become a clinical standard but is limited by the potential immunologic complications and availability of suitably matched donors. Gene therapy using autologous HSCs can avoid these limitations and thus may be safer.^[41]^ In this study, we attempted to identify the keywords most frequently listed in the past decade. Reference co-citations offers an informative snapshot of the network of publications and field of expertise in which numerous co-cited publications converge.^[12]^ Furthermore, clusters in the timeline view reveal the development of the research area.^[42,43]^ The timeline view visually reveals the historical span of the literature and is used to track the progression of research trends.^[44]^ Therefore, it is reasonable to conclude that “gene therapy” has been a topic of interest in recent years. Advances in gene editing can now offer potentially effective options for treating PIDs, which are mostly monogenic diseases.

In 2020, researchers demonstrated that <50% of patients requiring allogeneic HSCT had access to a suitably matched donor.^[45]^ Gene therapy using autologous HSCs, in which the patient own mutant HSCs are genetically modified, could avoid these limitations and has gained increasing momentum.^[46]^ Initially, Gene therapy started from the simple idea that replacing a disease-causing gene with healthy genes in HSC can cure the disease.^[47–49]^ The main drawback of the permanent gene addition approach is the unpredictability of the integration site of the therapeutic cassette. This leads to the inherent risk of insertional mutagenesis, oncogene transactivation and aberrant expression of the transgene and neighboring genes.^[50–54]^ Hence, researchers are searching for alternative approaches to gene therapy.

Our study provided links among the top productive authors, group authors and keywords. For example, the top productive author, Gennery AR, is a professor in Pediatric Immunology at Newcastle University (Top productive institutions). He is a member of European society for blood and marrow transplantation and European Society for Immunodeficiencies (Top grouped authors). His research focus on immunology in children, additionally, “chronic granulomatous disease” (Top keywords) was chosen for his research paper in 2018.^[37]^

Recently, clustered regularly interspaced short palindromic repeats (CRISPR)/CRISPR-associated protein (Cas)-based epigenetic, base, and prime editing systems have transformed the gene therapy landscape.^[46]^ CRISPR/Cas-based systems have the ability to recognize and bind to specific genomic sequences, thus enabling the correction of disease-causing mutations and site-specific disruption, and CRISPR/Cas-based systems avoid the inherent risk of insertional mutagenesis.^[55]^ Thus, CRISPR/Cas-mediated autologous HSCT is a prospective therapeutic option for different types of PIDs supported by preclinical gene-editing researches.^[56,57]^ Although genome editing with CRISPR/Cas system offers great promise for the treatment of human genetic diseases, it results in a mix of intended and unintended genetic alterations. In this manner, future work is needed to provide insights into more precise, more efficient and safer editing tools.^[58]^

## 5. Conclusion

We obtained deep insights into the treatment of PID using HSCT research through bibliometric analysis. The findings of this study will be helpful for researchers to understand the essential information in this field and to reveal the development of the research area.

## Author contributions

**Conceptualization:** Siqi Hu, Shixia Xu, Zhichun Feng, Xiangfeng Tang.

**Data curation:** Siqi Hu, Shixia Xu, Yi Wang.

**Formal analysis:** Siqi Hu, Wei Lu, Yingjian Si, Ya Wang, Zhenlan Du, Yi Wang, Xiangfeng Tang.

**Funding acquisition:** Zhichun Feng, Xiangfeng Tang.

**Investigation:** Zhichun Feng, Xiangfeng Tang.

**Methodology:** Wei Lu, Yingjian Si.

**Project administration:** Zhichun Feng, Xiangfeng Tang.

**Resources:** Siqi Hu, Shixia Xu, Ya Wang, Zhenlan Du.

**Software:** Siqi Hu, Shixia Xu.

**Writing – original draft:** Siqi Hu, Shixia Xu, Xiangfeng Tang.

**Writing – review & editing:** Siqi Hu, Shixia Xu, Zhichun Feng, Xiangfeng Tang.

## Supplementary Material


