# Supplementary material for: The research on the treatment of primary immunodeficiency diseases by hematopoietic stem cell transplantation: A bibliometric analysis from 2013 to 2022
Source: Medicine (Baltimore). 2023 Mar 31;102(13):e33295. doi: 10.1097/MD.0000000000033295 (PMC10063298; doi:10.1097/MD.0000000000033295)
Supplement: Supplementary file 1 [file medi-102-e33295-s001.pdf]

Table S1: The categories change during each year. Ratios are shown in parentheses.

2013	2014	2015	2016	2017	2018	2019	2020	2021	2022
Immunology (48.00)	Immunology (60.61)	Immunology (39.47)	Immunology (59.26)	Immunology (46.67)	Immunology (61.02)	Immunology (54.39)	Immunology (50.00)	Immunology (50.00)	Immunology (63.64)
Allergy (16.00)	Allergy (27.27)	Pediatrics (28.95)	Allergy (33.33)	Hematology (24.44)	Hematology (25.42)	Pediatrics (31.58)	Hematology (22.73)	Pediatrics (24.00)	Allergy (27.27)
Hematology (16.00)	Hematology (21.21)	Hematology (26.32)	Pediatrics (18.52)	Pediatrics (13.33)	Allergy (18.64)	Allergy (15.79)	Transplantation (15.15)	Hematology (20.00)	Pediatrics (18.18)
Pediatrics (16.00)	Oncology (18.18)	Allergy (15.79)	Hematology (11.11)	Allergy (11.11)	Transplantation (11.86)	Hematology (12.28)	Pediatrics (13.64)	Transplantation (12.00)	Endocrinology Metabolism (9.09)
Genetics Heredity (12.00)	Genetics Heredity (12.12)	Oncology (15.79)	Transplantation (11.11)	Transplantation (11.11)	Infectious Diseases (6.78)	Transplantation (8.77)	Allergy (12.12)	Allergy (8.00)	Hematology (9.09)
Pharmacology Pharmacy (12.00)	Medicine Research Experimental (12.12)	Genetics Heredity (7.89)	Infectious Diseases (7.41)	Genetics Heredity (8.89)	Pediatrics (6.78)	Oncology (7.02)	Medicine Research Experimental (6.06)	Medicine General Internal (6.00)	Infectious Diseases (9.09)
Transplantation (12.00)	Pediatrics (12.12)	Transplantation (7.89)	Medicine Research Experimental (7.41)	Medicine Research Experimental (8.89)	Multidisciplinary Sciences (5.08)	Surgery (5.26)	Oncology (6.06)	Oncology (6.00)	Pharmacology Pharmacy (9.09)
Biotechnology Applied Microbiology (8.00)	Biophysics (9.09)	Biotechnology Applied Microbiology (5.26)	Microbiology (7.41)	Biotechnology Applied Microbiology (6.67)	Medicine Research Experimental (3.39)	Dermatology (3.51)	Biotechnology Applied Microbiology (4.55)	Genetics Heredity (4.00)	Transplantation (9.09)

Medicine Research Experimental (8.00)	Transplantation (9.09)	Medicine General Internal (5.26)	Biophysics (3.70)	Infectious Diseases (6.67)	Biochemical Research Methods (1.69)	Infectious Diseases (3.51)	Infectious Diseases (4.55)	Gastroenterology Hepatology (2.00)
Biochemistry Molecular Biology (4.00)	Biotechnology Applied Microbiology (6.06)	Cell Biology (2.63)	Biotechnology Applied Microbiology (3.70)	Oncology (6.67)	Biochemistry Molecular Biology (1.69)	Microbiology (3.51)	Cell Biology (3.03)	Infectious Diseases (2.00)
Dentistry Oral Surgery Medicine (4.00)	Cell Biology (3.03)	Cell Tissue Engineering (2.63)	Genetics Heredity (3.70)	Pharmacology Pharmacy (4.44)	Biophysics (1.69)	Biophysics (1.75)	Education Scientific Disciplines (3.03)	Medicine Research Experimental (2.00)
Infectious Diseases (4.00)	Endocrinology Metabolism (3.03)	Critical Care Medicine (2.63)	Medicine General Internal (3.70)	Biochemical Research Methods (2.22)	Cell Biology (1.69)	Cell Biology (1.75)	Genetics Heredity (3.03)	Mycology (2.00)
Medicine General Internal (4.00)	Medicine General Internal (3.03)	Endocrinology Metabolism (2.63)	Oncology (3.70)	Cell Biology (2.22)	Dermatology (1.69)	Cell Tissue Engineering (1.75)	Multidisciplinary Sciences (3.03)	Nutrition Dietetics (2.00)
Microbiology (4.00)	Multidisciplinary Sciences (3.03)	Infectious Diseases (2.63)	Pathology (3.70)	Gastroenterology Hepatology (2.22)	Genetics Heredity (1.69)	Education Scientific Disciplines (1.75)	Biochemical Research Methods (1.52)	Virology (2.00)
Multidisciplinary Sciences (4.00)	Pathology (3.03)	Medicine Research Experimental (2.63)	Pharmacology Pharmacy (3.70)	Microbiology (2.22)	Microbiology (1.69)	Genetics Heredity (1.75)	Biochemistry Molecular Biology (1.52)	

Oncology (4.00)	Obstetrics Gynecology (2.63)	Tropical Medicine (3.70)	Multidisciplinary Sciences (2.22)	Oncology (1.69)	Medicine General Internal (1.75)	Biophysics (1.52)
	Pathology (2.63)			Pharmacology Pharmacy (1.69)	Medicine Research Experimental (1.75)	Cell Tissue Engineering (1.52)
	Pharmacology Pharmacy (2.63)			Surgery (1.69)		Clinical Neurology (1.52)
						Medicine General Internal (1.52)
						Pathology (1.52)
						Pharmacology Pharmacy (1.52)
						Rheumatology (1.52)
